# Perspectives on Immunoglobulins in Colostrum and Milk

**DOI:** 10.3390/nu3040442

**Published:** 2011-04-14

**Authors:** Walter L. Hurley, Peter K. Theil

**Affiliations:** 1 Department of Animal Sciences, University of Illinois at Urbana-Champaign, Urbana, IL 61801, USA; 2 Department of Animal Health and Bioscience, Aarhus University, DK-8830 Tjele, Denmark; Email: Peter.Theil@agrsci.dk

**Keywords:** immunoglobulins, milk, colostrum, bovine, human, immunity, passive transfer

## Abstract

Immunoglobulins form an important component of the immunological activity found in milk and colostrum. They are central to the immunological link that occurs when the mother transfers passive immunity to the offspring. The mechanism of transfer varies among mammalian species. Cattle provide a readily available immune rich colostrum and milk in large quantities, making those secretions important potential sources of immune products that may benefit humans. Immune milk is a term used to describe a range of products of the bovine mammary gland that have been tested against several human diseases. The use of colostrum or milk as a source of immunoglobulins, whether intended for the neonate of the species producing the secretion or for a different species, can be viewed in the context of the types of immunoglobulins in the secretion, the mechanisms by which the immunoglobulins are secreted, and the mechanisms by which the neonate or adult consuming the milk then gains immunological benefit. The stability of immunoglobulins as they undergo processing in the milk, or undergo digestion in the intestine, is an additional consideration for evaluating the value of milk immunoglobulins. This review summarizes the fundamental knowledge of immunoglobulins found in colostrum, milk, and immune milk.

## 1. Introduction

The topic of immunoglobulins in milk immediately brings to mind the relationship between mother’s milk, transfer of passive immunity from mother to neonate, and the immature immune system of the neonate. Research in this field dates back to the late nineteenth century, however for many centuries herdsmen have capitalized on the linkage between maternal immune status and the immunological protection and development of the neonate [1,2]. Immunoglobulins in mammary secretions come from several sources and represent a history of the antigen exposure of the mother and the response of her immune system. Immunoglobulins are transported through the mammary epithelial cells by receptor-mediated mechanisms and transferred out of the mammary gland by milk ejection during suckling. The immunoglobulins then enter the environment of the gastrointestinal tract of the neonate. Although that environment is primarily geared toward digestion to gain nutritional benefit, the immunoglobulins remain sufficiently stable to provide protective benefits for the neonate, either through uptake into the vascular system in the newborn of some species or through immunological function in the gastrointestinal tract. The immunoglobulins found in milk and the transfer of passive immunity from mother to neonate have been reviewed by many authors, with a partial listing referenced here [1,2,3,4,5,6,7,8,9,10,11,12,13,14,15,16,17,18].

In addition to the importance of homologous transfer of passive immunity between mother and neonate, there is considerable interest in the potential for heterologous transfer of passive immunity, such as immunoglobulins obtained from one species and utilized for passive immunity in another species. The ability to manipulate the immunological status of animals through vaccination against diseases that affect humans and the opportunity to harvest those immunoglobulins in the form of colostrum or milk has long been recognized [19,20], and continues to be a topic of interest in both animal science and human medicine [13,16,17,21,22,23].

This review begins with a summary of some of the research on what has been termed “immune milk” and then discusses various aspects of immunoglobulins in mammary secretions (structure, function, concentration, sources, transport, species differences, and roles of immunoglobulins). Finally, traits related to stability and processing methods for collecting milk immunoglobulins are reviewed.

## 2. Immune Milk

### 2.1. Overview

One intriguing application of our knowledge about bovine colostral and milk immunoglobulins comes through the opportunity to provide passive immunity against diseases in other species, especially in humans. The ability to direct the cow’s immune system to produce antigen-specific antibodies that are secreted in colostrum and milk and may be used to provide protection against a specific disease continues to be an area of interest. For example, the widespread consumption of immune milk from cows inoculated against diseases such as avian influenza, SARS, and other human respiratory diseases, has been suggested as a potential means of slowing outbreaks of the disease before they reach epidemic levels [24]. A number of reviews have summarized and evaluated early attempts to develop and test the use of immune milk products to provide passive immune protection [21,22,23,25,26,27,28,29,30,31]. Several immune milk products are available commercially [13,17,22,23,32]. Safety issues associated with use of bovine immune milk products for human use have been discussed by others [23,33,34,35]. The discussion below provides some examples of immune milk products and their use against some animal and human diseases (sections 2.2-2.7).

### 2.2. Homologous Transfer of Passive Immunity

Secretion of antibodies in breast milk from naturally immunized mothers can provide protection against enteric and other diseases in children [11]. For example, elevated concentrations of antibodies specific for enteric pathogens, such as *Vibrio cholerae*, in the mother’s breast milk do not prevent colonization with the bacterium in the nursing child, but do seem to protect the infected child from developing diarrhea [36]. Breast feeding is associated with a reduced incidence of Campylobacter diarrhea in young children compared with children that do not breast feed [37]. In those children that are breast fed and do develop diarrhea, the human milk consumed may not contain IgA antibodies specific for the common antigen of Campylobacter [37], suggesting a degree of antigen specificity contained in the breast milk.

The idea of immunizing the pregnant animal with the intent of controlling neonatal morbidity and mortality is well established [38]. Vaccination or natural immunization of the pregnant cow, ewe or sow against enterotoxigenic *Escherichia coli* [38,39,40] or intestinal viruses [41,42], can provide a degree of protection for the newborn. As an example, while only limited protection against viral challenge occurred in calves fed once shortly after birth with a pooled colostrum from cows immunized against bovine rotavirus, a shorter duration of diarrhea was observed [43]. On the other hand, calves fed milk supplements with low levels of a similar immune colostrum at each feeding for two weeks did have reduced virus sheading and reduced incidence of diarrhea [44].

In primates, immunization of pregnant baboons with a rhesus rotavirus vaccine increased milk immunoglobulin and virus neutralizing titre [45]. Prenatal immunization of pregnant women with a single dose of meningococcal vaccine not only increased antigen-specific IgG antibody in the infant’s serum during the initial 2-3 months after birth, but antigen-specific IgA in milk continued to be elevated at least up to 6 months [46]. As discussed in section 4, IgG transfer to the offspring in humans occurs during late pregnancy and provides the initial systemic source of that immunoglobulin. Infants consuming breast milk will primarily be consuming secretory IgA (section 4), which has significant protective activity in the intestine, as discussed in section 5.2.

### 2.3. Heterologous Transfer of Passive Immunity

The above examples of homologous transfer of passive immunity set the stage for considering the opportunities for heterologous passive transfer. Immune milk products generally are some form of protein product derived from the colostrum and/or milk of dairy cattle. The cows typically are hyperimmunized against one or more antigens representing pathogens of bacterial or viral origin. Crude preparations of the immunoglobulin from colostrum or milk may range from essentially no alteration of the immunoglobulin concentration in the product to partial immunoglobulin isolation or concentration in a whey protein concentrate.

The primary immunoglobulin in cow colostrum and milk is IgG, whereas the primary immunoglobulin in human milk is IgA [1]. Nevertheless, bovine IgG from colostrum or milk can be effective as a means of providing passive immunity to protect animals and humans from disease. The use of bovine colostral immunoglobulin preparations from immunized cows for disease protection of the neonate of other species has been demonstrated in swine [47], and experimental animal models such as mice [48,49]. There also are a number of examples of the use of bovine immune milk products in the treatment or prevention of human disease, especially in cases where the pathogen acts by way of the gastrointestinal tract. When considering these studies, it should always be kept in mind that the colostrum or milk preparations potentially contain other immune modulating substances than immunoglobulins, as discussed briefly below (section 6.3).

The concept of using immune milk derived from hyperimmunized cows for treatment of human disease can be traced back to the 1950s and earlier [19,20]. Some of the early efforts in this field involved using immune milk products for treatment of rheumatoid arthritis and hay fever [19]. Immune milk preparations produced from milk from cows immunized with a heat-killed, lyophilized mixture of bacteria found to reside in the human gastrointestinal tract has been studied for the prevention and treatment of rheumatoid arthritis, high blood cholesterol, high blood pressure, and oral submucous fibrosis [50,51,52,53]. On the other hand, most studies on the use of immune milk have examined the potential of immune milk for prevention and treatment of infectious diseases, particularly gastrointestinal disease.

Even milk that does not come form hyperimmunized cows may in some sense be regarded as immune milk. Bovine anti-human rotavirus IgG1 antibodies have been found in raw and pasteurized milk from cows that had not been specifically immunized against that virus [54]. Milk from non-immunized cows also has been found to contain measurable antigen-binding activity against several human pathogenic bacteria [55].

### 2.4. Immune Milk and Diseases Causing Diarrhea

Several authors have tested the efficacy of immunoglobulin preparations with antibody activity against human rotavirus as a means of providing passive immunity to children. For example, children consuming a defatted colostrum preparation from cows immunized against a strain of human rotavirus had no improvement of symptoms when the infection was established (patients admitted to a hospital with rotavirus infection), however the preparation was effective in limiting diarrhea in children when consumed prior to the infection [56,57]. In another study, cessation of excretion of rotavirus in the stool of infants with acute rotavirus gastroenteritis was correlated with the presence of neutralizing activity in the stool after ingestion of a bovine whey protein concentrate from rotavirus-hyperimmunized cows [58], although there was not a significant decrease in duration of diarrhea in that study. Other studies have found that treatment of children with hyperimmune bovine colostrum from cows immunized with human rotavirus serotypes reduces the duration and severity of diarrhea due to rotavirus [59], and can provide significant protection from rotavirus infection [60].

Enteropathogenic bacteria have also been the target for development of immune milk. Over 80% of childrens’ stools became negative for the *E. coli* strains used to hyperimmunize the cows that provided the source of immunoglobulin in a bovine colostrum/milk immunoglobulin concentrate consumed by children for 10 days [61]. Interestingly, only one in nine children treated with the immunoglobulin concentrate, and having diarrhea that was associated with *E. coli* strains which were not used in the immunization of the cows, developed negative stools, underscoring the importance of the bacterial strain-specificity of the immune product. Consumption of a hyperimmune immunoglobulin concentrate with a high antibody titer against a lipopolysaccharide isolated from *Shigella flexneri* 2a also has been shown to provide protection against a challenge with the same strain [62]. However, no difference in diarrhea or other symptoms in children with stools positive for *S. dysenteriae* was found whether treated with bovine colostrum from cows immunized against *S. dysenteriae* or with colostrum from cows not hyperimmunized [63].

Enterotoxigenic *E. coli* also is commonly associated with traveler’s diarrhea. Prophylaxis against this infection may be achieved by providing passive immunity with immune milk. A bovine whey protein concentrate from cows immunized with enterotoxigenic *E. coli* serotypes and consumed 3-times daily for seven days protected all of the adult volunteers from developing diarrhea after being challenged with an enterotoxigenic *E. coli* strain [64]. In contrast, 90% of the volunteers who received control immunoglobulin concentrate prior to challenge developed diarrhea after the *E. coli* challenge. Subsequent studies using IgG isolated from bovine colostrum from cows hyperimmunized against specific *E. coli* colonization factor antigens also have shown protective effects in volunteers challenged with colonization factor antigen-bearing enterotoxigenic *E. coli*[65], however other studies by the same group did not demonstrate significant effects of similar milk immunoglobulin products [66].

Bovine colostrum concentrate preparations derived from cows that have not been hyperimmunized against specific antigens also may provide some benefit via passive immunization for some diseases. For example, a commercial product which is made from large standardized pools of colostrum collected from over 100 cows has been used to treat a number of diseases [22,23], including diarrhea caused by diarrheagenic *E. coli* [67]. Similar preparations from non-immunized cows may provide protection against bacterial toxins that are the cause of diarrhea in AIDS patients [68]. These studies, along with the above mentioned study comparing colostrum preparations from cows immunized against *S. dysenteriae* or non-immunized cows [63], demonstrate that bovine colostrum contains significant antimicrobial properties as a result of natural exposure of the cows to antigens of pathogens that may afflict humans.

### 2.5. Immune Milk and Dental Caries

Another example of a potential use for bovine immunoglobulin preparations to control bacterial populations comes from studies on dental caries formation [69]. The concept of prenatal immunization of the pregnant mother to protect the neonate against dental caries was demonstrated in rats [70]. In applications to humans, bovine whey preparations of colostrum from cows immunized with caries-inducing bacterial strains (*Streptococcus mutans* and *Streptococcus sobrinus*), and containing over 60% immunoglobulin of which 80% was IgG1, has been used in several studies evaluating its effect on caries-producing bacteria. The colostral whey preparation reduced adherence of *Streptococcus mutans in vitro* and caused aggregation of suspended bacteria [71], as well as inhibited glucose uptake by the test organism [72,73]. The whey preparation from hyperimmunized cows opsonized bacteria and enhanced *in vitro* phagocytosis of bacteria by human leukocytes [74]. Antibodies in the whey preparation remained functional when added to milk that had been treated via ultra-high temperature pasteurization or milk that was fermented to extend shelf-life [75].

Immune milk from cows hyperimmunized against seven *Streptococcus mutans* strains reduced the recoverable bacterium in plaque samples from volunteers within seven days of initiation of mouth rinsing with the whey concentrate product [76]. Mouth rinsing with immune milk collected from cows immunized with a fusion protein representing two of the major factors implicated in oral colonization by *Streptococcus mutans* inhibited recolonization of saliva and plaque by that organism [77,78].

### 2.6. Immune Milk and Intestinal Parasites

Immunodeficiency disorders often are associated with cryptosporidiosis, which can lead to chronic malabsorption and weight loss. In a case study of a child with congenital hypogammaglobulinemia, severe vomiting and diarrhea due to cryptosporidiosis, gastric infusion with hyperimmune bovine colostrum from cows immunized with cryptosporidium oocytes resolved the symptoms within a few days and oocyts were no longer found in stool samples after about eight days [79]. Similarly, in a child with AIDS who had severe diarrhea caused by cryptosporidiosis, administration of a commercial hyperimmune bovine colostrum preparation with anticryptosporidial activity improved the diarrhea and eliminated the parasite [80].

### 2.7. Immunization to Boost Colostrum and Milk Antibodies

In the cases where immune milk is collected from cows immunized against one or more pathogens, the immunization regimen occurs during the prepartum period of the cow. To put this in perspective relative to the lactation cycle of a cow, a brief reminder of that cycle may be helpful. Depending on the management system used by a farm, most dairy cattle will have their first calf early in their third year, marking the start of their first lactation. The cow will be re-bred about two to three months into lactation. Pregnancy is approximately 280 days. At about 2 months before expected calving date, or approximately 10 months into lactation, milk removal is halted and the cow is given what is called a “dry” period. The mammary gland undergoes a process of involution during the early dry period where most residual milk components are broken down and resorbed [81]. The mammary gland begins a redevelopment phase several weeks prior to calving. Colostrum formation occurs in the days leading up to calving, coinciding with the early phase of lactogenesis (initiation of lactation). In the cow, lactogenesis begins shortly prior to calving and extends into the first few days postpartum. Colostrum collected at the first milking of the cow after calving represents the accumulation of colostral products during the days leading up to parturition, including immunoglobulins which are at their highest concentration in the first milking. Concentrations of immunoglobulins then decline rapidly in the subsequent several milkings [82].

One application for immunization of pregnant or lactating animals comes from the arena of mastitis control in cattle. Mastitis is the major disease in dairy cattle and most often is caused by intramammary infection [83,84]. Vaccination of cattle against mastitis-causing pathogens has been an area of study for many years [85,86]. Optimization of immunization schedules continues to be investigated [87]. Effective vaccines against mastitis-causing pathogens can increase antigen-specific immunoglobulins in the serum, which in turn can be increased in the mammary secretions. In the case of the J5 *E. coli* bacterin vaccine, the immunization also may be causing the mammary gland to become hyper-responsive to bacterial challenge [88], reminding us that enhancement of antigen-specific antibodies in the milk is not the only mechanism by which the vaccine may be having its effect.

Because the peripartum and early lactation periods are times of high susceptibility of the mammary gland to mastitis, many immunization schedules include prepartum immunizations during the dry period when milk is not removed and the mammary gland undergoes involution. It is also important to remember that cattle are generally immunosuppressed during the peripartum period [88,89], potentially compromising the impact of immunizations administered just before or just after calving.

Coliform mastitis is one of the major types of mastitis in cattle [90]. The more successful vaccination protocols for mastitis control have been with the J5 *E. coli* bacterin vaccine which is administered initially either just before or at the time of drying off [87,91,92,93,94,95,96]. These typically are followed by additional vaccine doses approximately mid-dry period. Some protocols include an additional immunization within several days after calving [87,91,92,94], while others also continue immunizations into the first three months of lactation [87,94]. Attempts to vaccinate against other mastitis-causing pathogens have been met with more limited success. Such vaccination protocols range from immunizations during the dry period [97], to peak lactation [98], and even late lactation [99]. Although most of the immunization protocols used in mastitis control administer the vaccine either intramuscularly or subcutaneously, intramammary immunization also can result in an increase in antigen-specific immunoglobulin in milk, as well as in the serum [100,101,102,103].

A look across the immunization protocols used in studies to produce many immune milk products shows considerable variation, especially in the number and timing of immunizations. In those specifically collecting colostrum shortly after calving, multiple immunizations are administered during late pregnancy when the cow would be in the dry period [56,57,58,60,61,64,71,72,77,79,104,105,106]. Mammary secretions then are collected either only at first milking [79], pooled from the first 4 to 6 milkings [56,57,72,104], pooled from the first 6 to 10 days after calving [58,61,64,105], or collected for longer periods into lactation [77]. Other studies have initiated immunizations during the late dry period and then continued vaccinating throughout lactation [50,51,76], or only vaccinating during lactation [107]. Many of these studies used intramuscular or subcutaneous immunization, although some also have incorporated intramammary [58,79,105], or intravenous infusion [61].

Newer technologies for vaccine development and delivery may further enhance the production of immune milk products. Immunization protocols that expose animals to specific antigens may enhance humoral immune responses in the mammary gland, including peptide-based vaccines [108], and DNA-based vaccines [109,110]. Delivery of antigen to the animal can also be achieved with antigen encapsulated in biodegradable microspheres [111], and with antigen-release devices [112]. Transgenic animals also have been used to produce antigens that then may be used to vaccinate animals against viral disease [113].

## 3. Immunoglobulin Structure and Function

The immunoglobulins, or antibodies, found in colostrum or milk are the same as those found in the blood or mucosal secretions. They are a family of proteins with a range of protective bioactivities. Immunoglobulins are divided into several classes including IgM, IgA, IgG, IgE, and IgD [114], and IgG, IgA and IgM are the major immunoglobulin classes in mammary secretions. IgM is the class that appears initially when an organism is exposed to an antigen for the first time (primary infection). IgM has a low specificity and hence a lower potency in defeating the infection. IgA is the major immunoglobulin class found in mucosal secretions and prevents mucosal infections by agglutinating microbes, whereas IgG is the primary immunoglobulin class found in bovine colostrum and milk. Several subclasses of IgG exist, with IgG1 and IgG2 being the major immunoglobulins in serum. All monomeric immunoglobulins have the same basic molecular structure, being composed of two identical heavy chains and two identical light chains, with a total molecular mass of approximately 160 kilodaltons (for details on immunoglobulin structure see [5,14,16]). Both the heavy and light chains have constant regions and variable regions. Heavy and light chains are linked together by disulfide bonds, resulting in the classic Y-shape of the immunoglobulin molecule [114]. The number and location of the disulfide bonds is dependent on the class of immunoglobulin. Each immunoglobulin molecule has two antigen binding sites which comprise the antigen-binding fragment (Fab). The Fab includes the variable amino acid domain. At the other end of the molecule is the constant fragment (Fc) which has a constant amino acid sequence among molecules of the same subclass and which confers the identity of an immunoglobulin as a particular subclass. The Fc region of the molecule is the region that binds to Fc receptors on various cell types.

In the case of polymeric immunoglobulins, including the polymeric forms of IgA and IgM that are found in milk, the monomeric forms of the immunoglobulins are linked together through the covalent interaction with a joining (J) chain [114,115]. The result is a dimeric form of IgA and a pentameric form of IgM. Binding of these immunoglobulins to the J chain also results in them having several special features, including: a high valency of antigen-binding sites, allowing them to agglutinate bacteria; limited complement-activating activity, which allows them to act in a noninflammatory manner; and a high affinity for the polymeric immunoglobulin receptor (pIgR) that is responsible for transepithelial transport of IgA and IgM into mucosal secretions such as milk [116]. The pIgR and its relationship to the secretory component (SC) associated with secretory IgA and secretory IgM is discussed further below (section 5.2). 

## 4. Concentrations in Colostrum and Milk-Physiological Conditions

The content of immunoglobulins in colostrum and milk is highly dependent on the animal species [1,14]. The same holds for the relative proportion of the immunoglobulin classes. These species differences are adaptations to the reproductive strategies of the animals and the degree of maturation of the offspring at birth. Animal species may be divided into three classes [1]: (1) species where immunoglobulins are transferred mainly to the fetus via the placenta (humans and rabbits); (2) species where offspring are born agammaglobulinemic and immunoglobulin transmission occurs via mammary secretions (ungulates such as horses, pigs, cows, and goats); and (3) species where immunoglobulins are transferred both via placenta and mammary secretions (rats, mice and dogs).

**Figure 1:**
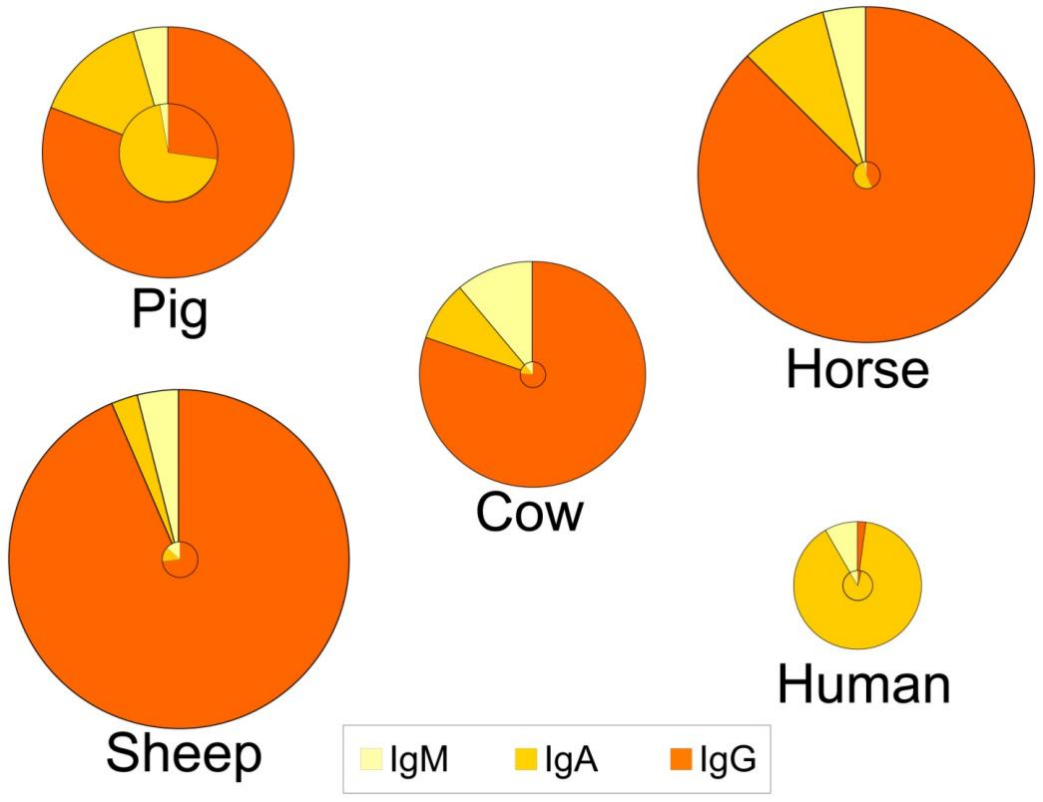
Relative distribution of IgG, IgA and IgM in colostrum (outer circle) and in milk (inner circle) of five species. The relative size of the circles represents the overall concentration of total immunoglobulins found among the species and the concentrations in colostrum *vs.* milk. Data compiled and calculated from: cow and sheep [1]; human and pig [3]; and horse [117].

These adaptations have several consequences both for the composition of immunoglobulins in colostrum and milk, and for the role of colostrum. Indeed, for animals like rats, mice, dogs and ungulates, uptake of colostrum of adequate quality and sufficient quantity is important for the offspring to boost the systemic immune function in the short term, whereas colostrum consumption in the human infant provides more protection for the gastrointestinal tract (see section 6.3). This is reflected in a lower total immunoglobulin content in human colostrum as compared to colostrum from the other species (Figure 1) [1,3,117]. Human colostrum has a low content of IgG (2%), and the IgG required to provide systemic immunity is transferred across the placenta before birth. In contrast, colostral IgG content in many other species is typically greater than 75% of the total immunoglobulin content (Figure 1). An additional consequence of different routes of immunoglobulin transmission relates to the changes in relative contents of immunoglobulins that occur in the transition from colostrum to milk within certain species (Figure 1). For example, the profile of immunoglobulins in human colostrum is similar to that found in milk, where the IgA level is high in both colostrum and milk (88-90% of total immunoglobulin). This is in contrast to the bovine mammary secretions where the high concentration of IgG in colostrum declines rapidly with successive milkings. For animals like rats, mice, dogs and ungulates, the role of colostrum and milk immunoglobulins is to provide immune protection both systemically and for the gastrointestinal tract, which is reflected in large changes in the profile of immunoglobulins during the transition from colostrum to mature milk (Figure 1). Thus, for many species the proportion of IgA increases between colostrum and milk.

## 5. Sources and Transport of Immunoglobulins

### 5.1. IgG

Immunoglobulins found in mammary secretions arise from systemic and local sources. In the case of IgG in milk, the major portion comes from the serum [14]. While IgG producing plasma cells may occur within the mammary tissue, their contribution to the IgG in colostrum is minor compared with the IgG derived from the serum.

Although limited paracellular passage of immunoglobulins may occur during inflammation (mastitis), uptake and transport of immunoglobulin across the mammary epithelial barrier is thought to occur primarily through an Fc-receptor-mediated process [1,7,118,119,120]. Immunoglobulins are thought to bind to receptors at the basolateral surfaces of the mammary epithelial cell. These receptors are specific for the Fc portion of the immunoglobulin molecule. The receptor-bound immunoglobulin is internalized via an endocytic mechanism [121], transported to the apical end of the cell and released into the alveolar lumen. Recent studies have shed additional light on the details of this process [122].

In the case of IgG, the receptor responsible for transcytosis of IgG into colostrum is referred to as FcRn, or the neonatal Fc receptor, because it was initially identified in the neonatal rodent intestine as the receptor responsible for the specific uptake of maternal IgG [123,124]. The FcRn also has been implicated in the trans-placental transport of IgG in humans and other species [125,126,127], which may involve an endocytic and transcytotic process [128]. Since its initial discovery, FcRn has been described in many tissues [122]. The receptor is a heterodimer composed of a membrane-bound α-chain similar to MHC class-1 molecules and a smaller MHC class-1 protein, β2-microglobulin [129]. Binding of IgG to FcRn is pH-dependent, with high affinity binding occurring at acidic pH, but only weak binding at neutral or basic pH [122]. This observation suggests that IgG taken up by the epithelial cells may bind to FcRn within an acidic environment in the endosomes. The precise mechanism of transport across the epithelial cell and release into the colostrum or milk remains to be demonstrated.

The half life of IgG in serum is typically longer (1-3 weeks) than that for IgA or IgM (1-2 days), and the half-life of IgG2 is slightly longer than for IgG1 [122]. Evidence suggests that IgG2 has a higher affinity for FcRn than IgG1 [122]. In bovine colostrum, IgG1 is many fold greater in concentration than IgG2 [82], although they are of approximately equal concentrations in serum. It may be that the majority of the IgG2 taken up by the mammary epithelial cell during colostrum formation is not passed on to the alveolar lumen, but rather is recycled back to the extracellular fluid. The FcRn is thought to have a major role in the recycling of IgG in various tissues in the body [130,131,132]. That is, IgG that potentially may be lost through various tissues is recycled by the respective cells by binding to FcRn and recycled back to the blood or lymph. This is supported by studies of overexpression of FcRn in transgenic mice where there is an extension of the half-life of serum IgG [133,134], as well as a boosting of the overall humoral immune response of the mice [135].

Localization of FcRn in bovine, sheep and water buffalo mammary tissue indicates that the receptor is homogeneously distributed throughout the epithelial cells prior to parturition, but primarily localized at the apical surface of the mammary epithelial cells after parturition [136,137,138,139]. While this type of observation corroborates the conclusion that FcRn plays an important role in IgG transport during colostrum formation, at least in ruminant species, the precise meaning of this redistribution of FcRn staining in mammary cells remains to be determined. It is also interesting to note that the initial report of this distribution pattern in sheep mammary epithelium included the observation that the staining pattern became diffuse within the cells during mammary involution [136,137]. Transport of IgG also may increase transiently in mammary secretions during involution in cattle [140]. Hormonal and local factors have been implicated in the control of immunoglobulin transport during colostrum formation [32].

Haplotypes of the *FCGRT* gene, coding for the MHC Class I α-chain of FcRn, are associated with serum concentrations of IgG in neonatal beef calves [141] and associated with IgG concentrations in colostrum of dairy cows [142]. Haplotypes of the β2-microglobulin gene (β2M) also are associated with serum IgG concentrations in newborn calves [143]. In estimating mass transfer of IgG1 into colostrum in dairy cattle, 10% of cows had mass transfer greater than one standard deviation above the mean, perhaps indicating a genetic or hormonal regulation of the variance of transport [144]. Clearly there is opportunity for genetic manipulation of IgG transport in the mammary gland to enhance the concentrations of immunoglobulins in colostrum and milk. However, it should be remembered that serum IgG concentrations in the periparturient cow are already decreased as a result of the extensive IgG transport into the colostrum [145], and as indicated above, the cow is in an immunosuppressed state during the peripartum period [88,89].

### 5.2. Secretory IgA and IgM

The other major classes of immunoglobulins transported into colostrum and milk are IgA and IgM. Immunoglobulin A is the major immunoglobulin in human colostrum and milk (Figure 1), however it is also present in milk of most other species. Colostrum and milk IgA and IgM are found in the form of secretory IgA, or sIgA, and sIgM. Much of these are produced by plasma cells in the mammary tissue. The plasma cells are part of the gut-associated lymphoid tissue (GALT), the largest immune organ of an organism, which includes the Peyer’s patches, lymphoid and myeloid cells in the *lamina propria* and intraepithelial lymphocytes [146,147]. Lymphocytes from the GALT system migrate to the mammary gland and provide a direct link between the antigen exposure response in the mother’s mucosal immune system, especially via the enteric mucosal immune system, and the secretory immunoglobulin repertoire of the mammary gland [18]. This means that maternal colostrum and milk will contain antibodies specific for pathogens that may be encountered by the neonate’s intestine and other mucosal tissues [10,18,148], providing a rationale for the observations summarized above that bovine colostrum from nonimmunized cows also may afford passive immune protection against human pathogens [54,55].

The immune connection between the GALT and the mammary gland is of particular interest with respect to human milk where the major immunoglobulin is sIgA, which accounts for one of the key factors underlying the importance of breast feeding [10]. The immune activation of GALT in the human infant is delayed, and the milk sIgA and sIgM provide the neonatal intestine a level of protection through their immune exclusion actions and their anti-inflammatory effects [10,18]. 

Transepithelial transport of IgA and IgM across the mammary epithelial cells occurs via the polymeric immunoglobulin receptor (pIgR) which is responsible for binding dimeric IgA and pentameric IgM in mucosal tissues [149,150]. The polymeric nature of IgA and IgM arises from their binding with the J-chain peptide [116]. Only IgA or IgM that contain the J chain have a high affinity for pIgR [116,151,152]. In fact, the J chain has been evolutionarily conserved within tetrapods to the point where human polymeric IgA can bind to the pIgR from the amphibian *Xenopus laevis* [152]. Polymeric IgA or IgM bound to pIgR is internalized and transported to the apical end of the mammary epithelial cell by an endocytic process. The pIgR molecule is cleaved to release a receptor fragment, called secretory component (SC), which remains bound to the immunoglobulin molecule [119,149]. In the case of pIgR receptor sites that are not occupied by immunoglobulin, the secretory component is still cleaved from the membrane-bound portion of pIgR, resulting in release of free secretory component. The secretory component has protective effects of its own, potentially blocking epithelial adhesion of enterotoxigenic *E. coli* and neutralizing the effects of other pathogens [148].

Expression of pIgR in the mammary gland is under control of hormones responsible for initiation of lactation [153]. Elevated transport of IgA also may occur during mammary gland involution in cattle and persist longer into the involution process [140].

## 6. Role of Immunoglobulins in the Intestine

### 6.1. Uptake of Immunoglobulins

Part of the transfer of passive immunity story in mammals involves the timing and location of transfer of immunoglobulins from the mother to the offspring, while another part encompasses the fate and function of the immunoglobulins once in the neonate [1,7,127]. In humans, intestinal transfer of maternal IgG from colostrum is sparse in the neonate and their immune competency is assured by transfer via the placenta. In rats and mice, there is FcRn-mediated uptake of IgG from the colostrum and milk in the neonate intestine. In ungulate species such as cattle, sheep, goats and pigs, the young are born essentially agammaglobulinemic and rely entirely on uptake of colostral immunoglobulins, especially IgG, for systemic immune protection. 

The consumption of colostrum by the neonatal calf has significant effects on the gastrointestinal tract [154]. The intestinal uptake in the immediate period after birth is transient and nonselective in species such as cattle, sheep, goats, swine and others. The intestinal cells become unable to absorb macromolecules within 24-36 h after birth probably as a result of developmental processes occurring in the enterocytes [155]. The process whereby the intestinal cells gradually stop absorbing macromolecules is termed “closure”. Before closure, the enterocytes will nonselectively absorb large molecular weight proteins and other molecules [155]. Macromolecules so transported are released into the *lamina propria* and then are absorbed into the lymphatic or portal circulation. Failure of passive transfer of immunity in these species is defined as occurring when a threshold concentration of IgG is not reached before closure occurs, which in the calf corresponds to serum IgG levels less than 10 mg/mL [156]. The maternal IgG in the calf’s blood gradually declines over the initial month after birth, and has a half-life of approximately 16 days [157].

### 6.2. Immunoglobulin-Intestinal Interactions

Milk sIgA is not taken up by the infant’s intestinal mucosa [148,158]. In fact, gut closure in humans occurs before birth and little immunoglobulin is absorbed intact in the intestine after birth [148,158]. However, the presence of sIgA in the intestinal lumen is part of the protective function of the epithelial barrier in the intestine [159]. Milk sIgA in the intestine will bind bacteria, toxins and other macromolecules, limiting their ability to bind to intestinal cells and thereby be transported through the mucosa to the *lamina propria* to cause a systemic immune response [160]. In adults of a pIgR-deficient strain of mice, which do not transport sIgA into the intestinal lumen, there is an increased serum IgA and IgG that react with commensal organisms and food antigens [161]. This may be occurring because sIgA is not being secreted into the intestinal lumen to participate in its role in immune exclusion (see section 6.3), and resulting in an increased uptake of food antigens and microbial antigens from the intestinal lumen which pass to the *lamina propria* and stimulate specific antibody responses [161]. Development of the GALT system is dependent on microbial stimulation [148,158]. The microbe binding function of sIgA then modulates the early microbial colonization of the gastrointestinal tract and the interaction of those microbes with the developing neonatal immune system [148,158,160].

### 6.3. Intestinal Actions of Colostrum and Milk Immunoglobulins

From the discussion of immune milk products above it was clear that these products have protective effects on neonatal health, as well as infant and adult human health. The exact mechanisms by which immune milk products have their effects are less clear and deserve further investigation. Below are summarized several perspectives to consider when evaluating the effects of immune milk products and the role of immunoglobulins in achieving those effects.

It should be remembered that colostrum and milk not only contain immunoglobulins, but also contain a range of antimicrobial factors and factors that may impact the immune system [10,154,160,162,163,164,165,166,167,168]. These include the iron-binding antimicrobial protein lactoferrin, antibacterial enzyme lactoperoxidase, antibacterial and lytic enzyme lysozyme, oligosaccharides that function as analogues of microbial ligands on mucosal surfaces, antimicrobial heat stable peptides (defensins), and soluble CD14. In addition, colostrum and milk contain leukocytes, including activated neutrophils, macrophages and lymphocytes. Colostrum also contains cytokines and growth factors that may affect neonatal intestinal development, as well as intestinal immune responses to disease in adults [166,169]. The relative concentrations of these factors vary considerably among species. Furthermore, colostrum provides a source of energy which may impact IgG absorption in the neonate [170], and provide additional energy for an effective immune response.

Another point to consider is that, while most macromolecules are degraded by digestive enzymes, some portion of macromolecules is transported across the intestine intact, including proteins [171,172]. Much of the immunoglobulin consumed in an immune milk can be expected to be partially or completely digested (discussed in section 7.3), however some portion of the immunoglobulin will remain intact or at least partially intact and capable of binding to an antigen.

All colostrum and milk will contain some sIgA, even those collected from cattle. The sIgA present in these secretions may contribute to the protective effects of immune milk products. Secretory IgA is considered to be the primary immunoglobulin responsible for immune protection of mucosal surfaces such as the intestine [158]. Secretory IgA and sIgM, as polymeric forms of the respective immunoglobulins, are stabilized by their binding to SC. They have antimicrobial properties such as agglutination of microbes and neutralization of viruses, and noninflammatory extracellular and intracellular immune exclusion by inhibiting adherence and invasion of mucosal epithelia [158]. The intracellular immune exclusion occurs when sIgA is being transcytosed by the enterocytes and comes into contact with viral particles within the endosomic system [15]. Secretory IgA also neutralizes pathogens in the intestinal lumen [173]. Bacterial enterotoxins may be neutralized by binding sIgA and internalization into intestinal epithelial cells [174]. 

In addition, IgA has a major role in the immunosuppressive mechanisms in the intestine that inhibit proinflammatory responses to oral antigens, which is part of the oral tolerance mechanisms in the intestine [158]. This suppression of the proinflammatory mechanisms is counterbalanced by systemic immune factors, including systemic IgG, which may result in inflammation and tissue damage once an antigen crosses epithelia barrier to the *lamina propria* [158]. 

After closure, any IgG localized in the *lamina propria*, whether from systemic sources or from uptake from the intestinal lumen, could contribute to proinflammatory responses in the intestine [158]. Indeed, post-closure uptake of IgG can occur via the FcRn receptor. FcRn has been identified in the human adult intestine [175,176], consistent with the hypothesis that FcRn is involved in IgG recycling (discussed in section 5.1). However, the transport of IgG across the enterocyte seems to be bidirectional, lending support to the concept that IgG in the intestine is involved in immune surveillance and defense of the mucosal lining [176,177,178]. Intestinal FcRn may deliver IgG-antigen immune complexes to the *lamina propria* for immune processing [158,177], thereby enhancing local mucosal immune response. 

On the other hand, functionally intact IgG that remains in the intestinal lumen might be expected to bind antigens and participate in protection of the tissue through immune exclusion. The intestinal mucus layer does provide an important protective barrier in the interactions of the intestinal tissue with microbes [179]. Interestingly, an IgG Fc binding site has been identified in association with the intestinal mucus [180,181,182]. This IgG Fc binding protein is distinct from the FcRn receptor. The Fc binding protein may block passage of IgG-antigen complexes to the enterocyte surface, thereby blocking their uptake and transport to the *lamina propria*, and perhaps allowing the complexes to be degraded in the intestinal lumen and excreted [169,182].

Consumed colostrum also may impact immunological development of the neonate [1]. These maternal antibodies may then inhibit infant responses to vaccine administration and impact development of the infant’s immunity [183].

## 7. Immunoglobulin Isolation and Stability

### 7.1. Overview

In the case of dairy animals producing colostrum or milk immunoglobulins for human consumption, immunoglobulins are harvested at milking and undergo various types of processing whether it is to prolong the shelf-life of the milk, to concentrate or isolate the immunoglobulins from the mammary secretion, or to digest the milk in the intestine. Through such processing, immunoglobulins are exposed to a number of conditions that may alter the structure and function of the protein. Some of methods used to concentrate or isolate the immunoglobulins include steps that involve exposing the protein to heat, acid or pressure which may affect the conformation of the protein, and ultimately the immunological activity of the antibody.

### 7.2. Isolation of Immunoglobulins from Mammary Secretions

A range of methods have been used for isolation of immunoglobulins from colostrum or milk. These include traditional methods of ammonium sulfate precipitation and column chromatography [3,5,145,184]. Affinity chromatographic methods used to isolate IgG include lectins [185]; protein A or G chromatography [186,187], and more recently, isolation with protein A/G immobilized electrospun polyethersulfone membranes [188]; metal chelate chromatography [189,190]; and adsorption with polyanhydride microparticles [191]. The range of detection and quantification methods for IgG, most often analyzed by radial-immunodiffusion [192] or enzyme-linked immunosorbant methods [193], are now expanding to include methods that detect multiple proteins, such as thermally addressed immunosorbant assays [194], and rapid methods that may be integrated into milking systems, such as surface plasmon resonance-based immunosensors [195]. 

### 7.3. Effects of Digestive Enzymes

Pepsin is a major proteolytic enzyme produced by the stomach. Pepsin digestion of IgG yields an F(ab’)_2_ fragment that includes the two antigen-binding (Fab) sites of the IgG molecule [5,114,196,197]. Intact immunoglobulin, F(ab’)_2_ and other antibody formats are being exploited in development of antibody therapeutics [198]. 

In the small intestine, immunoglobulins are further digested by pancreatic enzymes. One of them, trypsin, preferentially digests bovine IgG1 over IgM, whereas another enzyme, chymotrypsin, preferentially hydrolyzes IgM over IgG [199]. Bovine IgG1 is more susceptible to hydrolysis by pepsin than IgG2, while IgG2 is more susceptible to trypsin [200].

Immunoglobulins are relatively more resistant to gastrointestinal digestion than other milk or colostral proteins. Upon ingestion and entry into the stomach, the caseins form a curd under the influence of the acidic environment and proteolytic activity. As a consequence, casein is retained in the stomach of the neonate longer than the whey proteins, including IgG [201]. In the intestine, the fate for the other major whey proteins is rapid digestion for α-lactalbumin, while β-lactoglobulin is more slowly digested. Intestinal digestion of IgG is among the slowest of the whey proteins and IgG provides the smallest proportion of amino acids to the neonate relative to the other major whey proteins [201]. *In vitro* incubations of IgA and IgG with small intestinal content of young lambs have shown that IgA is more resistant towards digestion than is IgG [17]. 

In adult humans consuming a bovine whey protein concentrate, approximately 59% of IgG and IgM was detected by radial immunodiffusion from effluents from the jejunum, while 19% was detected in the ileum [202]. These estimates of digestion of immunoglobulin compare with estimates of digestion of milk proteins in adult humans which are approximately 42% complete at the end of the jejunum and 93% complete by the end of the ileum [203], again underscoring the relative resistance of immunoglobulins to digestion in the gastrointestinal tract. Detectable immunoglobulin in stool samples of infants fed the same immune product accounted for 10% of the ingested immunoglobulin [58]. In adults fed a bovine immunoglobulin concentrate, fecal IgG was typically less than 4% of ingested dose [204]. Detectable IgG in the stool [204], or ileal effluent samples of adults [205], is not significantly increased by prior treatment with a proton pump inhibitor to reduce stomach acid production. However, encapsulation of the immunoglobulin product can significantly increase the IgG detectable in the stool [204], although only low levels of IgG are detectable in the ileum of adults ingesting encapsulated immunoglobulin [205]. These studies suggest that degradation of immunoglobulins is occurring throughout the intestinal tract [202].

The primary structure of the immunoglobulin found in intestinal effluents most likely is the immunoglobulin Fab or F(ab’)_2_ fragments found in their stool [58,202], which nevertheless maintains its antigen-binding activity, as indicated by the correlation between appearance of the immunoglobulin and the virus-neutralizing activity observed in stool samples [58]. In adults ingesting bovine anti-*Clostridium difficile* immunoglobulins, toxin-neutralizing activity paralleled the bovine IgG content in ileal effluent [205], and in stool samples [204]. A pepsin-resistant form of bovine IgG representing approximately 10% of colostral immunoglobulin has been isolated with a lectin that binds *O*-linked oligosaccharides [185], indicating that some proportion of IgG in the gastrointestinal tract may remain intact.

### 7.4. Effects of pH

The pH of bovine mammary secretions transiently drops at calving (to approximately pH 6.4), then increases over several days to pH 6.6 to 6.9 [206], which is the pH characteristic of mature milk. Therefore, bovine colostrum is slightly more acidic than mature milk. Studies of isolated immunoglobulin stability over a pH range indicate that bovine IgG isolated from milk is stable for several hours at 37 °C when in pH 6-7, however stability is significantly reduced at pH ≤ 3 and ≥10 [207,208]. The negative effect of pH on IgG stability, even in the range of 4.5-6.5, is enhanced under elevated temperature conditions [209,210]. The use of a multiple emulsion to encapsulate milk IgG may increase stability of the protein against extreme acidic or alkali conditions, as well as against proteolytic degradation [211]. However, emulsification by homogenization may reduce the residual IgG content of the emulsion product [211], probably as a result of high shear forces [208]. Ultrasonic treatment of isolated IgG also decreases residual IgG content [208].

### 7.5. Effects of Heat Treatment

Immunoglobulins are thermolabile. Exposure to temperatures of 75 °C can reduce detectable isolated bovine IgG by 40% in 5 min, and by 100% at 95 °C for 15 s [208]. Heat exposure causes conformational changes in the IgG molecule [212]. Antigen-binding activity of bovine IgG also is reduced after heat treatment [209,213]. This is consistent with studies that suggest that the antigen-binding region of the immunoglobulin molecule is more thermolabile than the other regions of the molecule [209,214]. Detectable IgG in colostrum or colostral whey also are reduced by heat treatment, however at a slower rate than for isolated IgG. Thermal protectants such as sugars or glycerol can increase the stability of isolated IgG to heat treatment [208,215]. 

Many milk processing protocols include heat treatment of the colostrum, milk or whey. Of the major immunoglobulin classes in bovine milk, IgG is the most thermostable and IgM is the least thermostable [214]. Commercial milk samples that have undergone a typical pasteurization process, including skim milk powder, can retain 25-75% of the IgG concentration compared with raw milk, while milk undergoing ultra-high temperature (UHT) pasteurization contains little detectable IgG [192,216]. Nevertheless, antigen-specific IgG in milk is relatively stable under typical conditions of pasteurization when compared with that in UHT milk or cow milk-based infant formulas that undergo high-temperature processing [54,217]. 

Flash-heat treatment of human breast milk, a method recommended by WHO to reduce vertical transmission of HIV in resource-poor regions, has minimal effects on milk IgA and antimicrobial activity of the milk [218,219]. This method involves placing a jar of milk into a water bath, the water bath is heated to boiling, and then the jar of milk is removed and allowed to cool. The milk reaches a maximum temperature of 72-73 °C and is above 56 °C for over 6 min [218,219].

Alternative methods of achieving microbial inactivation may offer a means of avoiding the impact of heat treatment on IgG solutions. For example, high voltage pulsed electric fields have been used as a nonthermal processing method for pasteurization in various foods [220,221,222]. Pulsed electric field processing also generates heat, however temperature exposure of the fluid is less than 50 °C, and total treatment time exposure is in milliseconds [223]. That compares with more typical pasteurization process at about 72 °C for 2 min. Microbial inactivation in bovine IgG solutions as a result of pulsed electric fields did not change the secondary structure or the thermal stability of the secondary structure of the IgG [224], and antigen-binding activity was unchanged [223]. Another emerging technology that may provide a nonthermal microbial inactivation treatment for milk uses exposure to pulsed ultraviolet light [225].

High-pressure processing is another non-thermal method with the potential for inactivation of microbial and certain enzymes in food products, thereby extending shelf-life of the product [226]. While the high-pressure process also generates heat during the treatment of the sample, lowering the initial temperature of the sample allows for control of the maximum temperature reached to be maintained within a desired range [227]. To be effective in inactivating bacterial spores, high-pressure processing needs to be combined with moderate temperature treatment [228]. Moderate to extensive loss of immunoactivity of IgG may occur depending on the conditions used for high-pressure processing of colostrum or other IgG-containing fluids [227,229]. High-pressure processing also has been used for human breast milk with minimal effect on the milk IgA [230].

### 7.6. Heating Colostrum

The issue of heating effects on immunoglobulin and colostrum also is important for control of various diseases that occur in cattle. Collection and storage of colostrum from dairy cows shortly after calving has long been a common procedure. The stored colostrum then is fed to newborn calves to assure adequate uptake of IgG for protection of the calf. Several pathogens can be transmitted from cow to calf via colostrum or milk [231]. Colostrum may contain these pathogens as a result of shedding from the mammary gland, contamination of the colostrum after harvesting or improper storage of colostrum prior to feeding calves [231]. One approach to allowing the neonate the benefits of colostrum from infected cows is collecting colostrum and batch pasteurization of pooled colostrum prior to feeding to the calves [232]. Volume of the batch of pooled colostrum that is pasteurized affects measurable IgG concentrations in the colostrum, as well as IgG serum concentrations attained in calves after feeding the colostrum [232]. Heat treatment of colostrum at 60 °C for one to two hours does not alter measurable IgG concentrations or viscosity of the colostrum, nor does that treatment affect antibody activity [231,233]. In addition, bacteria inoculated into colostrum prior to a heat treatment of 60 °C for one hour are not detectable after the heat treatment [234]. On-farm heat treatment of colostrum (60 °C for one hour) results in higher concentrations of serum IgG and greater apparent absorption efficiency of IgG in new born calves consuming the treated colostrum than consumption of raw colostrum [235,236,237].

## 8. Conclusions

Colostrum and milk are rich sources of immunoglobulins. These secretions have developed through evolution to ensure homologous transfer of passive immunity from mother to offspring. The immunoglobulins that are passed from mother to her offspring, whether by transplacental transfer or by ingestion of colostrum and milk, can form an important link between the immunological experience of the mother and the immune capacity of the newborn. This immunological link also includes many immune factors that may be present in mammary secretions other than the immunoglobulins. The immunoglobulins in colostrum and milk also provide opportunities to harness their immunological function for the benefit of other animals, including humans. Research has demonstrated that bovine colostrum and milk, whether or not they are from cows immunized against specific pathogens, provide a medium for the heterologous transfer of passive immunity, and may offer disease protection in a range of species. New technologies for enhancing efficacy of vaccination, enhancing stability and extending shelf-life of the immunoglobulin preparation while minimizing the impact of the processing, and extending the effectiveness of the immunoglobulin in the intestine, may enhance future use of colostrum and milk based on their potent immunological activity. While the mechanisms by which immunoglobulins are transferred from mother to neonate and their role in the neonate have become well documented, additional research is needed to clarify the mechanisms of action of the immunoglobulins derived from milk or colostrum when used in animals that are developmentally more mature.
